# Validity and diagnostics of the Reading the Mind in the Eyes Test (RMET) in non-demented amyotrophic lateral sclerosis (ALS) patients

**DOI:** 10.3389/fpsyg.2022.1031841

**Published:** 2022-11-02

**Authors:** Edoardo Nicolò Aiello, Laura Carelli, Federica Solca, Silvia Torre, Roberta Ferrucci, Alberto Priori, Federico Verde, Vincenzo Silani, Nicola Ticozzi, Barbara Poletti

**Affiliations:** ^1^IRCCS Istituto Auxologico Italiano, Department of Neurology and Laboratory of Neuroscience, Milan, Italy; ^2^Ph.D. Program in Neuroscience, School of Medicine and Surgery, University of Milano-Bicocca, Monza, Italy; ^3^Aldo Ravelli Center for Neurotechnology and Experimental Brain Therapeutics, Department of Health Sciences, International Medical School, University of Milan, Milan, Italy; ^4^ASST Santi Paolo e Carlo, San Paolo University Hospital, Milan, Italy; ^5^IRCCS Ca’ Granda Foundation Maggiore Policlinico Hospital, Milan, Italy; ^6^“Dino Ferrari” Center, Department of Pathophysiology and Transplantation, Università degli Studi di Milano, Milan, Italy

**Keywords:** Reading the Mind in the Eyes Test, amyotrophic lateral sclerosis, executive, diagnostics, psychometric

## Abstract

**Background:**

The aim of this study was to explore the construct validity and diagnostic properties of the Reading the Mind in the Eyes Test (RMET) in non-demented patients with amyotrophic lateral sclerosis (ALS).

**Materials:**

A total of 61 consecutive patients and 50 healthy controls (HCs) were administered the 36-item RMET. Additionally, patients underwent a comprehensive assessment of social cognition *via* the Story-Based Empathy Task (SET), which encompasses three subtests targeting Causal Inference, Emotion Attribution (SET-EA), and Intention Attribution (SET-IA), as well as global cognitive [the Edinburgh Cognitive and Behavioral ALS Screen (ECAS)] and behavioral screening [the Frontal Behavioral Inventory (FBI); the Dimensional Apathy Scale (DAS); the Beck Depression Inventory (BDI); and the State and Trait Anxiety Inventory-Y]. The construct validity of the RMET was tested by regressing it within a stepwise model that encompassed as predictors the abovementioned cognitive and behavioral measures, covarying for demographic and motor confounders. Receiver-operating characteristics (ROC) analyses allowed exploring intrinsic and post-test properties of the RMET both in discriminating patients from HCs and in identifying patients with a defective SET-EA performance.

**Results:**

The RMET was solely predicted by the SET-EA (*p* = 0.003) and SET-IA (*p* = 0.005). RMET scores showed high accuracy both in discriminating patients from HCs (AUC = 0.81) and in identifying patients with a defective SET-EA score (AUC = 0.82), with adequate-to-optimal both intrinsic and post-test properties.

**Discussion:**

The RMET is a convergently and divergently valid measure of affective social cognition in non-demented ALS patients, also featuring optimal intrinsic and post-test diagnostic properties in both case-control and case-finding scenarios.

## Background

Due to the pathophysiological and genetic link between amyotrophic lateral sclerosis (ALS) and frontotemporal degenerations, up to 50% of non-demented patients with ALS happen to show mild-to-moderate, frontotemporal-like cognitive deficits–the most prominent involving executive and language functioning–whose psychometric evaluation is clinically pivotal given their renown, detrimental impact on patients’ prognosis (Strong et al., 2017).

Deficits of social cognition, i.e., those sets of cognitive processes which allow to represent and process of socially relevant and emotional stimuli in order to enact adaptive behaviors within interpersonal relations (Arioli et al., 2018), are also acknowledged to feature the cognitive profile of non-demented ALS patients (Bora, 2017; Carelli et al., 2021)–with their detection being sufficient, according to Strong et al.’s (2017) revised *consensus* criteria, to classify them as cognitively impaired. More specifically, deficits in emotion processing and recognition, as well as in the ability to represent and attribute others’ mental states, appear to be typical of ALS patients’ cognitive phenotype (Bora, 2017; Strong et al., 2017; Carelli et al., 2021). Considering the ecological relevance of social-cognitive functioning, which underpins adaptive behaviors in several everyday-life scenarios (Arioli et al., 2018; Maresca et al., 2020), as well as the overall negative impact of cognitive dysfunction on patients’ prognoses (Huynh et al., 2020), the availability of statistically sound, and standardized tests to assess social cognition in ALS patients is clinically crucial.

In this respect, recent meta-analytic evidence (Taule et al., 2020) suggests that the Reading the Mind in the Eyes Test (RMET)–a widespread measure of visual, non-verbal emotion recognition, and mental state attribution (Baron-Cohen et al., 2001)–is, among all domain-specific, second-level tests for assessing cognition in patients with ALS, the one that received the strongest clinimetric support. Moreover, by simply requiring participants to determine which one of four words best describes the emotion expressed by a pictured eye region (Baron-Cohen et al., 2001), the RMET is untimed and accommodates motor disabilities (i.e., dysarthric patients can deliver their responses by pointing, while those with upper-limb impairments do so by verbalizing them), making the test highly feasible in this population (Taule et al., 2020). However, no study has to date explored the diagnostic properties of the original, 36-item RMET in ALS patients (Taule et al., 2020)–such analyses have only been performed on a shortened, and less widespread, form (Burke et al., 2020). Moreover, as it is being debated whether socio-cognitive deficits merely arise from cognitive/behavioral dysexecutive features (van der Hulst et al., 2015; Watermeyer et al., 2015; Burke et al., 2016a,^2017^; Arioli et al., 2018) or are, at least to some extent, independent of them in this population (Girardi et al., 2011; Trojsi et al., 2016; Palumbo et al., 2022; Panopoulou et al., 2022), little is known on the construct validity of tests supposedly targeting social cognition in patients with ALS, including the RMET (Maddaluno et al., 2021).

Given the pivotal relevance of delivering evidence about the clinimetric value of cognitive tests in order to increase their level of recommendation in clinical practice and research (Taule et al., 2020), this study aimed at exploring the construct validity and diagnostic properties of the RMET in patients with ALS.

## Methods

### Participants

A total of 61 consecutive ALS patients referred to IRCCS Istituto Auxologico Italiano, Milano, Italy, between 2016 and 2022 and 50 healthy controls (HCs) were recruited. Exclusion criteria, applying to both groups, were the following: (1) (further) neurological or psychiatric diagnoses; (2) severe general-medical conditions; and (3) uncorrected hearing/vision deficits. ALS was diagnosed by means of El Escorial revised criteria (Brooks et al., 2000). No patient met the current criteria for behavioral variant-frontotemporal dementia (Rascovsky et al., 2011) or primary progressive aphasia (Gorno-Tempini et al., 2011). The study was approved by the Ethics Committee of IRCCS Istituto Auxologico Italiano (I.D.: 2013_06_25); participants provided informed consent, and data were treated according to current regulations.

### Materials

Both groups were administered the Italian version of the original RMET (maximum score achievable: 36) (Maddaluno et al., 2021). The Italian RMET has been shown to be featured by adequate internal and test-retest reliability and construct validity, as well as being underpinned by a mono-factorial structure (Serafin and Surian, 2004; Vellante et al., 2013; Preti et al., 2017). Additionally, patients underwent a comprehensive assessment of social cognition *via* the Story-Based Empathy Task (SET; maximum score achievable: 18) (Dodich et al., 2015), which encompasses three subtests targeting causal inference (SET-CI; maximum score achievable: 6), emotion attribution (SET-EA; maximum score achievable: 6), and intention attribution (SET-IA; maximum score achievable: 6), as well as global cognitive (the Edinburgh Cognitive and Behavioral ALS Screen; ECAS) (Poletti et al., 2016) and behavioral screening [the Frontal Behavioral Inventory (FBI) (Alberici et al., 2007); the Dimensional Apathy Scale (DAS) (Santangelo et al., 2017); the Beck Depression Inventory (BDI) (Beck et al., 1961); and the State- and Trait-Anxiety Inventory-Y (STAI-Y1/STAI-Y2) (Spielberger et al., 1971)]. Motor-functional outcomes were evaluated *via* the ALS Functional Rating Scale-Revised (ALSFRS-R) (Cedarbaum et al., 1999), King’s staging system (Roche et al., 2012), and progression rate (ΔFS)–the latter being computed as (48-ALSFRS-R total score/disease duration in months) (Kimura et al., 2006). Neuropsychological assessments were performed by psychologists/neuropsychologists (FS, LC, and ST), while neurological examinations by neurologists (NT and VS)–both classes of practitioners having long-lasting expertise in ALS care.

### Statistics

Receiver-operating characteristics (ROC) analyses were run to derive intrinsic–i.e., sensitivity (Se) and specificity (Sp)–and post-test diagnostic properties–i.e., positive and negative predictive values (PPV and NPV) and likelihood ratios (LR+ and LR−)–at the optimal cutoff identified *via* Youden’s *J* statistic. Demographically adjusted RMET scores (Maddaluno et al., 2021) were entered into the case-control ROC analysis (i.e., when addressing the occurrence of ALS as the positive state), since the two groups were matched for education but not for age–the latter having been identified as a significant confounder of the RMET according to the Italian norms herewith adopted (Maddaluno et al., 2021). To further support the validity of such a ROC analysis, as well as in order to rule out a potential effect of sex, for which the two groups were unmatched, an *F*-test was preliminarily run on RMET raw scores by covarying for age, education, and sex and including a Sex*Group interaction term. RMET diagnostics within a case-finding setting were instead tested, within the patient cohort, by addressing RMET raw scores against age- and education-adjusted, below-cutoff performance on the SET-EA (Dodich et al., 2015).

Since RMET raw scores distributed normally [i.e., skewness and kurtosis values < |1| and |3|, respectively (Kim, 2013)], its construct validity was tested, within the patient cohort, by regressing it within a stepwise multiple linear models, encompassing as predictors SET (SET-CI/-EA/-IA), ECAS (ECAS-Language/-Fluency/-Executive/-Memory/-Visuo-spatial), and behavioral scores (FBI, BDI, and STAI-Y1/-Y2) and covarying for demographic (age, education, and sex) and motor confounders (disease duration, ALSFRS-R bulbar, respiratory and upper-/lower-limb subscores, and ΔFS). Significant predictors were selected by applying Bonferroni’s correction (α_*adjusted*_ = 0.05/number of target predictors, i.e., excluding covariates).

Sample size estimations for this study were performed by addressing the most relevant set of analyses, i.e., ROC ones, through *easyROC*.^1^ The minimum sample size for a case-control ROC analysis was set at *N* = 48, with an allocation ratio of 1 [i.e., patients with ALS (*N* = 24) and HPs (*N* = 24)] and by addressing the following parameters: AUC = 0.7, α = 0.5, and 1–β = 0.8. As to the case-finding ROC analysis, by forecasting, based on Consonni et al. (2016), a prevalence of ≈13% of patients with ALS performing defectively on the SET-EA (i.e., an allocation ratio of 8), the minimum sample size was set at *N* = 54 (i.e., patients with a defective SET-EA score [*N* = 6] and performing normally [*N* = 48])–with an AUC = 0.8, α = 0.5, and 1–β = 0.8.

Analyses were run using R 4.1^2^ and jamovi 2.3 (the jamovi project, 2022); missing values were excluded pairwise, and the significance level was set at 0.05.

## Results

Table 1 shows the background and clinical measures of participants. The prevalence of age- and education-adjusted, below-cutoff RMET scores (Maddaluno et al., 2021) was 0% in HCs and 4.9% in patients.

**TABLE 1:** Background and cognitive measures of participants.

	ALS	HCs	*p*
*N*	61	50	–
Age (years)	62.1 ± 11.3 (28–82)	51.9 ± 11.4 (36–75)	<0.001^a^
Sex (M/F)	52.5%/47.5%	32%/68%	0.030^a^
Education (years)	12.4 ± 4.1 (5–18)	13 ± 4 (5–19)	0.45^b^
Disease duration (months)	19.8 ± 21.3 (2–108)	–	–
**ALSFRS-R**			
Total	40 ± 6.4 (22–48)	–	–
Bulbar	10.4 ± 2 (6–12)	–	–
Spinal–lower limbs	11.9 ± 3.7 (2–16)	–	–
Spinal–upper limbs	6.1 ± 2.1 (0–8)	–	–
Respiratory	11.5 ± 1 (7–12)	–	–
ΔFS	0.78 ± 0.99 (0–5.3)	–	–
**KSS**			
Stage 0	6.7%	–	–
Stage 1	35.6%	–	–
Stage 2	28.9%	–	–
Stage 3	26.7%	–	–
Stage 4	2.2%	–	–
PEG	1.6%	–	–
NIV	0%	–	–
**Genetics**			
*C9orf72*	1.6%	–	–
*SOD1*	1.6%	–	–
*TARDBP*	1.6%	–	–
**ECAS**			
Total	101.8 ± 16.6 (43–127)	–	–
ALS-specific	75.2 ± 13.7 (34–95)	–	–
ALS-non-specific	26.7 ± 4.8 (9–33)	–	–
Language	23.9 ± 3.5 (15–28)	–	–
Fluency	17.5 ± 5 (0–24)	–	–
Executive	33.6 ± 7.2 (15–45)	–	–
Memory	15.3 ± 4.3 (1–21)	–	–
Visuo-spatial	11.4 ± 1.3 (6–12)	–	–
**RMET**			
Raw scores	22.2 ± 4.2 (10–30)	27.1 ± 3 (20–34)	<0.001^c^
Below-cutoff scores^d^	4.9%	0%	
SET	12.3 ± 4.1 (1–18)	–	–
SET-CI	4.2 ± 1.6 (0–6)	–	–
SET-EA	3.7 ± 1.7 (0–6)	–	–
SET-IA	4.2 ± 1.6. (0–6)	–	–
FBI	2.7 ± 2.7 (0–12)	–	–
DAS	22.5 ± 7.4 (3–40)	–	–
BDI	13.5 ± 9 (0–37)	–	–
STAI-Y1	55.3 ± 12.1 (34–87)	–	–
STAI-Y2	51 ± 9 (38–73)	–	–

ΔFS, progression rate; ALS, amyotrophic laterals sclerosis; ALSFRS-R, Amyotrophic Lateral Sclerosis Functional Rating Scale-Revised; BDI, Beck Depression Inventory; CI, Causal Inference; DAS, Dimensional Apathy Scale; EA, Emotion Attribution; ECAS, Edinburgh Cognitive and Behavioral ALS Screen; F, female; FBI, Frontal Behavioral Inventory; IA, Intention Attribution; KSS, King’s staging system; M, male; NIV, non-invasive ventilation; PEG, percutaneous endoscopic gastrostomy; SET, Story-Based Empathy Task; STAI-Y1, State and Trait Anxiety Inventory–Form Y–State Anxiety; STAI-Y2, State and Trait Anxiety Inventory–Form Y–Trait Anxiety.

^a^χ^2^-statistic (test of independence); ^*b*^*t*-statistic; ^*c*^*F*-statistic (covaried for age, education, and sex); ^d^ Maddaluno et al. (2021).

At α_*adjusted*_ = 0.025, RMET raw scores were solely predicted by the SET-EA (β = 0.44; *p* = 0.003) and SET-IA (β = 0.41; *p* = 0.005), with 50.1% of their variance being explained by such a model [*F*(2,31) = 15.54; *p* < 0.001].

The preliminary analysis of covariance on raw RMET scores revealed that patients with ALS performed worse than HCs [*F*(1,105) = 28.5; *p* < 0.001; η^2^ = 0.21] net of age education and sex–with the former two covariates predicting the outcome (*p* < 0.001), at variance with sex (*p* = 0.406) and the Sex*Group interaction (*p* = 0.311).

**FIGURE 1:**
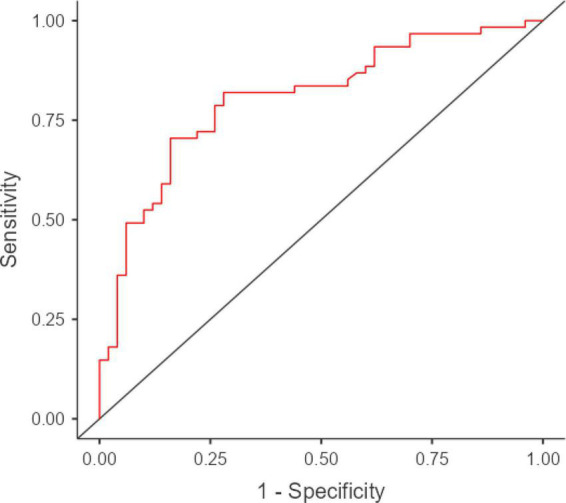
ROC curve for RMET-adjusted scores in discriminating patients with ALS from HCs. ROC, receiver-operating characteristics; RMET, Reading the Mind in the Eyes Test; ALS, amyotrophic lateral sclerosis; HCs, health controls. AUC = 0.81; SE = 0.04; 95% CI [0.77, 0.92]. Visualization was performed using jamovi 2.3 (https://www.jamovi.org/) by means of the *R* package *ROCR* (https://cran.r-project.org/package=ROCR).

Reading the mind in the eyes test-adjusted scores showed high accuracy in discriminating patients from HCs (AUC = 0.81; SE = 0.04; 95% CI [0.77, 0.92]) (Figure 1), with optimal both intrinsic (SE = 0.71; Sp = 0.84) and post-test properties (PPV = 0.84; NPV = 0.7; LR+ = 4.41; LR− = 0.35) at the optimal cutoff (<24.977; *J* = 0.55).

**FIGURE 2:**
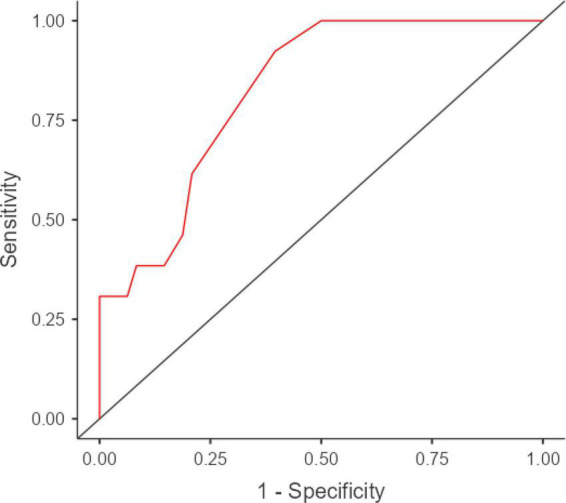
ROC curve for RMET raw scores in discriminating patients with ALS having a below- vs. above-cutoff SET-EA scores. ROC, receiver-operating characteristics; RMET, Reading the Mind in the Eyes Test; ALS, amyotrophic lateral sclerosis; SET-EA, Story-Based Empathy Task-Emotion Attribution. AUC = 0.82; SE = 0.06; 95% CI [0.77, 0.93]. Visualization was performed using jamovi 2.3 (https://www.jamovi.org/) by means of the *R* package *ROCR* (https://cran.r-project.org/package=ROCR).

Similarly, when aiming to identify patients with a below-cutoff performance on the SET-EA (21.3%), RMET raw scores high accuracy (AUC = 0.82; SE = 0.06; 95% CI [0.77, 0.93]) (Figure 2), as well as optimal intrinsic features (SE = 0.92; Sp = 0.6), in spite of suboptimal post-test diagnostics (PPV = 0.39; NPV = 0.97; LR+ = 2.33; LR− = 0.13) at the optimal cutoff (<23; *J* = 0.53).

## Discussion

This study provides, for the first time, strong evidence on the validity of the RMET as a social-cognitive measure in non-demented patients with ALS, demonstrating its diagnostic soundness in both case-control and case-finding scenarios.

Within the patient cohort, the RMET indeed proved to be independent of behavioral dysexecutive features, including apathy, as well as from anxiety and depression levels, diverging from measures of executive functioning or of other cognitive domains (language, memory, and visuo-spatial abilities). At variance, only the SET-EA and SET-IA, which target mental state attribution abilities (Serafin and Surian, 2004), were found to be associated with RMET scores. Most importantly, such an association was not found with the SET-CI, which, by contrast, assesses one’s general ability to draw causal inferences. Taken together, such findings strongly support the convergent and divergent validity of the RMET as a selective measure of affective social cognition in non-demented ALS patients, especially when such evidence yields regardless of motor confounders.

Such results are remarkable, as, for the first time, they show that a social-cognitive measure can be simultaneously disentangled from behavioral status, executive functions, and other cognitive domains in this population–in line with previous studies (Girardi et al., 2011; Trojsi et al., 2016; Palumbo et al., 2022; Panopoulou et al., 2022) but, at the same time, in contrast with others (van der Hulst et al., 2015; Watermeyer et al., 2015; Burke et al., 2016a,^2017^). Indeed, Watermeyer et al. (2015) and Burke et al. (2016a) found that social-cognitive functioning was prominently affected by executive measures in ALS, while van der Hulst et al. (2015) and Burke et al. (2017) showed that patients with ALS with social-cognitive impairment presented with prominent behavioral changes. At variance, Girardi et al. (2011) suggested that social-cognitive deficits and dysexecutive features can be dissociable in ALS, while Trojsi et al. (2016), Panopoulou et al. (2022), and Palumbo et al. (2022) found associations between social-cognitive and non-executive cognitive measures (i.e., memory, language, or visuo-spatial skills). In this respect, at variance with such investigations (Girardi et al., 2011; van der Hulst et al., 2015; Watermeyer et al., 2015; Burke et al., 2016a,^2017^; Trojsi et al., 2016; Palumbo et al., 2022; Panopoulou et al., 2022), this study has also the merit of regressing a social-cognitive measure by concurrently accounting for both executive and non-executive cognitive functioning, behavioral status, motor confounders, and demographic background, thus warranting an adequate degree of external and ecological validity for the findings herewith reported. Nevertheless, it has to be borne in mind that these results are measure-dependent–i.e., are to be referred to the RMET only: while this test herewith appears to “purely” target social cognition in non-demented patients with ALS, the same might not apply to other social-cognitive measures. After all, it is indeed theoretically and empirically reasonable (Abrahams, 2011; Maresca et al., 2020; Carelli et al., 2021) to postulate that, in this population, social-cognitive abilities can be, at the same time, both linked and independent of executive/non-executive cognitive functions and behavioral status, as in fact suggested by the present finding of 49.9% of the variance of the RMET being unexplained by SET-EA/-IA scores.

With further regard to the regression model herewith run, it has to be noted that, in line with a recent report (Palumbo et al., 2022), RMET scores were found to be unrelated to measures of disease severity and progression. Relatedly, this study does not support the notion of bulbar involvement being a risk factor for lower RMET scores, at variance with what previous evidence showed (Burke et al., 2016b). Thus, findings herewith reported further avail the notion of the RMET being highly feasible and not biased by motor disabilities in this population (Strong et al., 2017; Taule et al., 2020).

With regard to diagnostic efficiency analyses on the RMET, the present work supports its ability to accurately differentiate HCs from non-demented ALS patients, being sound as to both its intrinsic and post-test features. A similar argument applies to the capability of the RMET to identify patients presumably presenting with mental state attribution deficits (as operationalized by a defective SET-EA performance)–albeit, in such a case-finding scenario, the test proved to be featured by a poor PPV. However, one should note that predictive values are prevalence-based diagnostic properties, this meaning that the present finding of a poor PPV of the RMET within the case-finding scenario might have been biased by a relatively low prevalence of the target condition (i.e., a defective SET-EA).

This study is, of course, not free of limitations. First, although such an issue has been managed statistically, it should be mentioned that HCs were younger than patients with ALS at a group level, as well as that women were overrepresented in HCs, at variance with an optimal male/female ratio within the ALS cohort. Thus, further investigations are advisable that replicate the present findings by comparing fully matched samples as far as demographics are concerned. Moreover, with specific regard to the ALS cohort, it has to be mentioned that patients were in relatively early stages of the disease; this prevents generalizing the present findings on the association between RMET scores and motor confounders to patients with more advanced, and thus possibly severe, disease stages. Moreover, this work only addressed non-demented patients with ALS; thus, further studies are desirable that compare this population to patients also presenting with co-morbid frontotemporal dementia (Strong et al., 2017). As to the psychometric instruments herewith addressed, it should be also noted that further research is needed to assess the construct validity of the RMET in this population against second-level, domain-specific cognitive measures other than the ECAS, which is, at variance, a screener. Finally, a statistical note is worth doing on the SET, namely that, within its original development and normative study (Serafin and Surian, 2004), no evidence of validity or reliability was provided, and future studies should test such clinimetric features in order to fully support the present findings. With that said, it should be likewise noted that the authors of the SET itself, as well as independent Italian researchers, had previously shown its feasibility and clinical usefulness in patients with ALS (Cerami et al., 2014; Crespi et al., 2016, 2020; Palumbo et al., 2022), thus supporting the adoption of such a task as a social-cognitive measure, at least on a clinical, practical level.

## Conclusion

This study supports the notion that the RMET is a valid measure of affective social cognition in non-demented patients with ALS and features by optimal intrinsic and post-test diagnostic properties in both case-control and case-finding scenarios. Thereupon, the findings herewith reported add up to and complement the existing literature on the feasibility of the RMET in this population, thus further availing its adoption within both clinical practice and research as addressed to non-demented patients with ALS.

## Data availability statement

The raw data supporting the conclusions of this article will be made available by the authors, without undue reservation.

## Ethics statement

The studies involving human participants were reviewed and approved by the Ethics Committee of IRCCS Istituto Auxologico Italiano (I.D.: 2013_06_25). The patients/participants provided their written informed consent to participate in this study.

## Author contributions

EA: conceptualization, analyses, drafting, and revision. FS: conceptualization, data collection, drafting, and revision. ST and LC: data collection and revision. RF and AP: revision. FV, VS, NT, and BP: conceptualization, resources, drafting, and revision. All authors contributed to the article and approved the submitted version.

## Footnotes

1
http://www.biosoft.hacettepe.edu.tr/easyROC/

2
https://cran.r-project.org/
